# A Novel Mutation in the *CYP11B1* Gene Causes Steroid 11*β*-Hydroxylase Deficient Congenital Adrenal Hyperplasia with Reversible Cardiomyopathy

**DOI:** 10.1155/2015/595164

**Published:** 2015-07-22

**Authors:** Mohammad A. Alqahtani, Ayed A. Shati, Minjing Zou, Ali M. Alsuheel, Abdullah A. Alhayani, Saleh M. Al-Qahtani, Hessa M. Gilban, Brain F. Meyer, Yufei Shi

**Affiliations:** ^1^Department of Pediatrics, Aseer Central Hospital, Abha 62523, Saudi Arabia; ^2^Department of Child Health, College of Medicine, King Khalid University, Abha 61421, Saudi Arabia; ^3^Department of Genetics, King Faisal Specialist Hospital and Research Centre, Riyadh 11211, Saudi Arabia

## Abstract

Congenital adrenal hyperplasia (CAH) due to steroid 11*β*-hydroxylase deficiency is the second most common form of CAH, resulting from a mutation in the *CYP11B1* gene. Steroid 11*β*-hydroxylase deficiency results in excessive mineralcorticoids and androgen production leading to hypertension, precocious puberty with acne, enlarged penis, and hyperpigmentation of scrotum of genetically male infants. In the present study, we reported 3 male cases from a Saudi family who presented with penile enlargement, progressive darkness of skin, hypertension, and cardiomyopathy. The elder patient died due to heart failure and his younger brothers were treated with hydrocortisone and antihypertensive medications. Six months following treatment, cardiomyopathy disappeared with normal blood pressure and improvement in the skin pigmentation. The underlying molecular defect was investigated by PCR-sequencing analysis of all coding exons and intron-exon boundary of the *CYP11B1* gene. A novel biallelic mutation c.780 G>A in exon 4 of the *CYP11B1* gene was found in the patients. The mutation created a premature stop codon at amino acid 260 (p.W260^*∗*^), resulting in a truncated protein devoid of 11*β*-hydroxylase activity. Interestingly, a somatic mutation at the same codon (c.779 G>A, p.W260^*∗*^) was reported in a patient with papillary thyroid cancer (COSMIC database). In conclusion, we have identified a novel nonsense mutation in the *CYP11B1* gene that causes classic steroid 11*β*-hydroxylase deficient CAH. Cardiomyopathy and cardiac failure can be reversed by early diagnosis and treatment.

## 1. Introduction

Congenital adrenal hyperplasia (CAH) is a group of autosomal recessive disorders caused by inactivating mutations in genes involved in the cortisol biosynthesis. More than 90% of CAH cases are due to steroid 21-hydroxylase deficiency. Steroid 11*β*-hydroxylase is encoded by* CYP11B1* and its deficiency is the second most common cause of CAH. At an incidence of one in 100,000 live births, it accounts for about 5 to 8% of cases of adrenal steroidogenic defects [1].

Cortisol is synthesized from cholesterol in the adrenal cortex involving five enzymatic steps: cleavage of the cholesterol side chain to yield pregnenolone, 3*β* dehydrogenation to progesterone, and successive hydroxylations at the 17*α*, 21, and 11*β* positions. Steroid 11*β*-hydroxylase catalyzes the final step of cortisol synthesis, converting 11-deoxycortisol and 11-deoxycorticosterone (DOC) to cortisol and corticosterone, respectively [2]. Mutations in the* CYP11B1* gene cause an impairment of these two reactions, resulting in excessive DOC accumulation and androgen production. Despite failure of aldosterone production, overproduction of DOC, a less potent mineralocorticoid, can bind and activate the mineralocorticoid receptor to cause salt retention and hypertension in about two-thirds of patients [1]. This clinical feature distinguishes 11*β*-hydroxylase deficiency from 21-hydroxylase deficiency in which the lack of DOC production and subsequently aldosterone causes renal salt wasting in most of the patients [3]. The excessive steroid precursors are shunted into the adrenal androgen synthesis pathway, resulting in virilization and ambiguous genitalia of genetically female infants. Postnatal androgen excess causes precocious pseudopuberty, rapid somatic growth, and accelerated bone maturation in both sexes. In addition, partial impairment of* CYP11B1* function leads to a milder, nonclassic form of steroid 11*β*-hydroxylase deficiency [4, 5] with a phenotype resembling to the nonclassic 21-hydroxylase deficiency [6]. However, unlike classic steroid 11*β*-hydroxylase deficiency, hypertension is not commonly found in the nonclassic form.

The* CYP11B1* gene is localized on chromosome 8q21, approximately 40 kb from the paralog* CYP11B2* gene which encodes aldosterone synthase [7]. Both* CYP11B1* and* CYP11B2* have 93% sequence similarity [7]. Chimeric* CYP11B1/CYP11B2* genes abolishing steroid 11*β*-hydroxylase function have also been described [8–10]. Based on The Human Gene Mutation Database (HGMD), there are 109 different mutations in the* CYP11B1* gene reported in the literature.

Although hypertension is common in steroid 11*β*-hydroxylase deficiency, cardiomyopathy due to long standing uncontrolled hypertension has less frequently been reported. Only three adults [11, 12] and two children [13, 14] have been reported in the literature. In the present study, we reported three additional cases and characterized the underlying genetic defect with discovery of a novel mutation in the* CYP11B1* gene.

## 2. Subjects and Methods

### 2.1. Case 1

A 30-month-old boy was admitted to the pediatric intensive care unit with heart failure and respiratory distress. He was found to have macropenis (penile length was 10 cm) and excessive skin darkness. His height was 99 cm and blood pressure at the presentation was 143/98 mmHg. Chest X-ray showed cardiomegaly and echocardiography showed severe dilated cardiomyopathy. The patient died at the third day of admission.

### 2.2. Case 2

**Figure 1:**
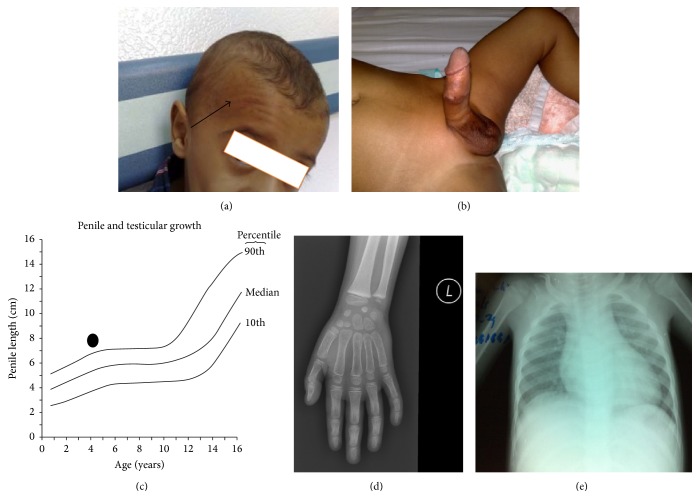
Clinical presentations of a patient with classic steroid 11*β*-hydroxylase deficiency. (a) Forehead showing facial acne indicated by a black arrow. (b) Macropenis. Penile is enlarged (8.7 cm in length) with scrotum hyperpigmentation. Testes volume is prepubertal and no pubic hair. (c) Age to penile length graph. The graph shows the penile length and growth (8.7 cm) and penile length is more than 90th percentiles. (d) Left hand X-ray shows advanced bone age between 5 to 6 years of age. (e) Chest X-Ray indicates mild to moderate cardiomegaly.

A 21-month-old boy was admitted to the emergency department with respiratory symptoms. He had history of progressive penile enlargement and darkness of skin for the last 10 months. His elder brother died 13 years ago with similar clinical presentations (Case 1). His weight was 13 kg (*Z*-score: 1.1, 85.3th percentile), height 92 cm (*Z*-score: 2.4, 99.2th percentile), and blood pressure 139/90 mmHg (*Z*-score for age-based pediatric blood pressure for systolic/diastolic pressure: 4.3/3.9, 100th percentile. *Z*-score equal to or greater than 95th percentile indicates hypertension). Physical examination showed gum, skin, and scrotal hyperpigmentation, facial acne, and penile enlargement of 8.7 cm (above 90th percentile). Testicular size was prepubertal (less than 2 mL) and no pubic hair (Figures 1(a), 1(b), and 1(c)). Laboratory tests showed serum ACTH: 507 pg/mL (normal: 5–60 pg/mL), cortisol: 44 *μ*g/dL (normal: 55–248 *μ*g/dL), 17*α*-hydroxyprogesterone (17-OHP): 67 nmol/L (normal: 0.3–2.5 nmol/L), DOC: 319 ng/dL (normal: 4–49 ng/dL), and androstenedione: 11 ng/mL (normal: 0.4–4.1 ng/mL). His bone age was at 5-6 years of age (Figure 1(d)). Chest X-ray showed mild to moderate cardiomegaly (Figure 1(e)). Echocardiography showed mild left ventricular dilatation with mild impairment of function with ejection fraction of 44% (normal 55–70%). The patient was diagnosed as congenital adrenal hyperplasia steroid 11*β*-hydroxylase deficiency based on his clinical presentations and lab tests. The patient was given hydrocortisone 50 mg (80 mg/kg/m^2^) intravenous bolus as a stress dose then followed by maintenance dose of 12.5 mg/kg/m^2^ (5 mg am, 2.5 mg noon, and 2.5 mg pm) with antihypertensive medication (captopril 6.25 mg orally three times a day). Six months later following treatment, echocardiography showed normal left ventricular systolic function. Blood pressure became normal at 88/44 mmHg (50 percentile according to his age). Hydrocortisone decreased to 2.5 mg orally three times a day as a maintenance dose to be continued for life. Gradual reduction of captopril was initiated from 6.25 mg once daily to complete cessation after 3 months. The skin color was improving and acne disappeared.

### 2.3. Case 3

His 10-month-old younger brother was evaluated due to having similar clinical presentations such as excessive skin darkening and progressive penile enlargement. His weight was 11 Kg (*Z*-score: 2.5, 99.4th percentile), height 88 cm (*Z*-score: 6.4, 99.9th percentile), and blood pressure 125/88 (*Z*-score: 3.6/3.8, 100th percentile). His penile was 7.5 cm (above 90th percentile) with scrotal hyperpigmentation and no pubic hair. Testicular size was less than 2 mL. His bone age was equivalent to 5-6 years old. Laboratory tests showed serum cortisol: 43.1 *μ*g/dL, androstenedione: 8.0 ng/mL, aldosterone: 11 pg/mL (20–1100 pg/mL), 17-OHP: 56 nmol/L, and DOC: 195 ng/dL. Echocardiography showed mild left ventricular dilatation with 47% ejection fraction. He was diagnosed as congenital adrenal hyperplasia steroid 11*β*-hydroxylase deficiency and treated with hydrocortisone 40 mg (80 mg/kg/m^2^) intravenous bolus as a stress dose then followed by maintenance dose around 12 mg/kg/m^2^ (2.5 mg am, 2.5 mg noon and 2.5 mg pm) with antihypertensive medication (captopril 6.25 mg orally three times a day). Follow-up echocardiography after 6 months treatment showed that dilated left ventricle was returned to normal with normal cardiac function (60% ejection fraction) and blood pressure was normal at 50% percentile. Same procedure as his elder brother was used for gradual reduction of hydrocortisone to maintenance dosage and eventual stop of captopril over 3 months.

The other siblings in the family (2 boys and one girl) had no signs of accelerated growth and sexual development, and their serum levels of deoxycorticosterone, 17-OHP, androgens, and aldosterone were within normal range, indicating that they were not affected with CAH.

### 2.4. Genomic DNA Isolation

Genomic DNA from peripheral blood leukocytes of patients was isolated using the Gentra Blood Kit (Qiagen Corp., CA) after informed consent was obtained from their parents. The parents and three unaffected siblings (two brothers and one sister) of the patients refuse to give blood samples for genetic test, which prevented us from performing a familial genetic analysis. The study was approved by our institutional review board.

### 2.5. DNA Amplification and Sequencing

Selective amplification of the* CYP11B1* gene was performed in five fragments by PCR from 100 ng of genomic DNA as described previously [11]. The resulting PCR products were directly sequenced using an automated ABI PRISM 3700 sequencer (Foster City, CA).

## 3. Results

### 3.1. Clinical Characteristics

The diagnosis of steroid 11*β*-hydroxylase deficient congenital adrenal hyperplasia was made based on their clinical and biochemical features. Both patients had characteristic features of classic steroid 11*β*-hydroxylase deficiency: accelerated growth, skeletal maturation, pseudoprecocious puberty, hypertension, elevated serum levels of DOC, 17-OHP, and androstenedione, and low serum levels of cortisol and aldosterone. The initial presentation of the patients was cardiac complications due to prolonged hypertension, which could result in misdiagnosis and miss early treatment opportunities as in the first patient who died due to heart failure. Hypertension with virilized genitalia and pseudoprecocious puberty should be alerted for the diagnosis of steroid 11*β*-hydroxylase deficiency.

### 3.2. Sequence Analysis of the* CYP11B1* Gene

**Figure 2:**
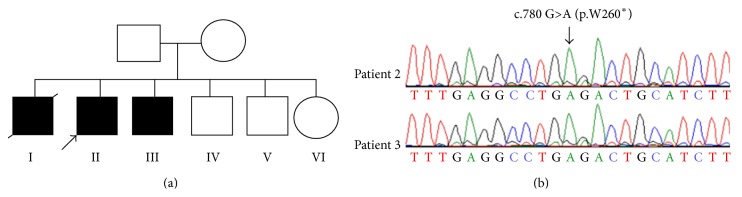
A novel nonsense mutation in the human* CYP11B1* gene. (a) Pedigree of a large Saudi family with* CYP11B1* gene mutation. Three brothers are diagnosed with steroid 11*β*-hydroxylase deficient congenital adrenal hyperplasia. One died at 30 months of age due to heart failure. Both parents and three siblings are unaffected. (b) Sequence analysis shows a biallelic c.780 G>A mutation in exon 4 of the* CYP11B1* gene in both patients from the family. The mutation creates a premature stop codon TGA. The mutation is indicated by an arrow. The parents refused to donate blood for genetic testing, but they are likely heterozygous carriers.

To identify the underlying genetic defects leading to steroid 11*β*-hydroxylase deficiency, we sequenced the entire coding region and intron-exon boundaries of the* CYP11B1* gene in both patients. A novel biallelic mutation in the* CYP11B1* gene was found in both patients. The mutation c.780 G>A created a premature stop codon at amino acid 260 (p.W260^*∗*^), resulting in functional inactivation of the* CYP27B1* gene (Figure 2). Interestingly, a somatic mutation at the same codon (c.779 G>A, p.W260^*∗*^) was reported in a patient with papillary thyroid cancer (COSMIC data base). The significance of the mutation in thyroid cancer remains to be determined. The parents and three unaffected siblings (two brothers and one sister) refused to donate blood samples for genetic test, which prevented us from performing a familial genetic analysis. However, the parents are likely heterozygous carriers.

## 4. Discussion

In the present study, we have presented three cases of classic steroid 11*β*-hydroxylase deficiency from nonconsanguineous parents. Their diagnosis was initially missed at the local hospital and the patients developed dilated cardiomyopathy due to hypertension. This could result in misdiagnosis and delay in early treatment as demonstrated in our first patient who died due to heart failure. Routine blood pressure measurements and a detailed physical examination during well baby visit can help detect underlying causes of hypertension in pediatric patients. Furthermore, a detailed physical examination should always include checking the genitalia which is often skipped by most general pediatricians during their busy practice. Hypertension with virilized genitalia and pseudoprecocious puberty such as infantile acne, macropenis, and darkness of skin should alert physicians for the diagnosis of steroid 11*β*-hydroxylase deficiency.

Hypertension occurs in approximately two-thirds of patients with classic steroid 11*β*-hydroxylase deficiency and it is most common early in life [1]. This is due to the accumulation of DOC as a consequence of inadequate 11*β*-hydroxylation in the biosynthesis of aldosterone. Hypertension is not common in patients with late-onset disease or nonclassic form of steroid 11*β*-hydroxylase deficiency due to partial 11*β*-hydroxylase activity [15]. The secondary hypertension leads to dilated cardiomyopathy in our patients, which can be reversed by early diagnosis and treatment. Since most reported cases are very young, it is not clear if there are any upper age limits after which the cardiac changes become irreversible despite adequate blood pressure control and CAH treatment.

The World Health Organization classifies cardiomyopathy into four categories: (1) dilated cardiomyopathy; (2) hypertrophic cardiomyopathy; (3) restrictive cardiomyopathy, and (4) arrhythmogenic right ventricular cardiomyopathy [16]. Among the four groups, dilated cardiomyopathy (DCM) is the most common in children [17–19]. DCM is a heterogeneous group of cardiac muscle disorders characterized by ventricular dilatation, impaired systolic function, and reduced myocardial contractility. The most common cause of dilated cardiomyopathy is an idiopathic etiology (>60%), followed by familial cardiomyopathy and acute myocarditis. Other causes of dilated cardiomyopathy include viral infections, endocrine disorders, and metabolic diseases. Cardiotoxic drugs and systemic diseases can also cause dilated cardiomyopathy. Genetic causes account for more than 30% of DCM cases [20]. In our three cases, only ventricular dilatation is found and it is likely a manifestation of cardiac failure due to ventricular dysfunction.

The nonsense mutation described in our patient has not been described in the literature. The mutated transcripts may be translated to a truncated protein or degraded via nonsense-mediated decay [21]. In either case, the 11*β*-hydroxylase activity is completely lost in our patients. The clinical presentation and lab data support the conclusion.

In summary, we have identified a novel nonsense mutation in the* CYP11B1* gene that causes classic steroid 11*β*-hydroxylase deficient CAH. Cardiomyopathy and cardiac failure can be reversed by early diagnosis and treatment.

## Abbreviations

CAH: Congenital adrenal hyperplasia
DOC: 11-Deoxycorticosterone
17-OHP: 17*α*-Hydroxyprogesterone.

## References

[1] White P. C., Curnow K. M., Pascoe L. (1994). Disorders of steroid 11*β*-hydroxylase isozymes. *Endocrine Reviews*.

[2] Nimkarn S., New M. I. (2008). Steroid 11*β*- hydroxylase deficiency congenital adrenal hyperplasia. *Trends in Endocrinology and Metabolism*.

[3] New M. I., Abraham M., Gonzalez B. (2013). Genotype-phenotype correlation in 1,507 families with congenital adrenal hyperplasia owing to 21-hydroxylase deficiency. *Proceedings of the National Academy of Sciences of the United States of America*.

[4] Joehrer K., Geley S., Strasser-Wozak E. M. C. (1997). CYP11B1 mutations causing non-classic adrenal hyperplasia due to 11*β*-hydroxylase deficiency. *Human Molecular Genetics*.

[5] Parajes S., Loidi L., Reisch N. (2010). Functional consequences of seven novel mutations in the CYP11B1 gene: four mutations associated with nonclassic and three mutations causing classic 11*β*-hydroxylase deficiency. *Journal of Clinical Endocrinology and Metabolism*.

[6] Speiser P. W., White P. C. (2003). Congenital adrenal hyperplasia. *The New England Journal of Medicine*.

[7] Mornet E., Dupont J., Vitek A., White P. C. (1989). Characterization of two genes encoding human steroid 11*β*-hydroxylase (P-450(11*β*)). *The Journal of Biological Chemistry*.

[8] Kuribayashi I., Nomoto S., Massa G. (2005). Steroid 11-beta-hydroxylase deficiency caused by compound heterozygosity for a novel mutation, p.G314R, in one CYP11B1 allele, and a chimeric CYP11B2/CYP11B1 in the other allele. *Hormone Research*.

[9] Portrat S., Mulatero P., Curnow K. M., Chaussain J.-L., Morel Y., Pascoe L. (2001). Deletion hybrid genes, due to unequal crossing over between CYP11B1 (11beta-hydroxylase) and CYP11B2 (aldosterone synthase) cause steroid 11beta-hydroxylase deficiency and congenital adrenal hyperplasia. *Journal of Clinical Endocrinology and Metabolism*.

[10] Hampf M., Dao N. T. N., Hoan N. T., Bernhardt R. (2001). Unequal crossing-over between aldosterone synthase and 11*β*-hydroxylase genes causes congenital adrenal hyperplasia. *The Journal of Clinical Endocrinology & Metabolism*.

[11] Chabre O., Portrat-Doyen S., Chaffanjon P. (2000). Bilateral laparoscopic adrenalectomy for congenital adrenal hyperplasia with severe hypertension, resulting from two novel mutations in splice donor sites of CYP11B1. *Journal of Clinical Endocrinology and Metabolism*.

[12] Hague W. M., Honour J. W. (1983). Malignant hypertension in congenital adrenal hyperplasia due to 11 beta-hydroxylase deficiency. *Clinical Endocrinology*.

[13] Al Jarallah A. S. (2004). Reversible cardiomyopathy caused by an uncommon form of congenital adrenal hyperplasia. *Pediatric Cardiology*.

[14] Bhatia S., Muranjan M. N., Lahiri K. R. (2012). Left ventricular failure due to a rare variant of congenital adrenal hyperplasia. *Indian Journal of Pediatrics*.

[15] de Simone G., Tommaselli A. P., Rossi R. (1985). Partial deficiency of adrenal 11-hydroxylase. A possible cause of primary hypertension. *Hypertension*.

[16] Richardson P., McKenna R. W., Bristow M. (1996). Report of the 1995 World Health Organization/International Society and Federation of Cardiology Task Force on the definition and classification of cardiomyopathies. *Circulation*.

[17] Arola A., Jokinen E., Ruuskanen O. (1997). Epidemiology of idiopathic cardiomyopathies in children and adolescents. A nationwide study in Finland. *The American Journal of Epidemiology*.

[18] Lipshultz S. E., Sleeper L. A., Towbin J. A. (2003). The incidence of pediatric cardiomyopathy in two regions of the United States. *The New England Journal of Medicine*.

[19] Nugent A. W., Daubeney P. E. F., Chondros P. (2003). The epidemiology of childhood cardiomyopathy in Australia. *The New England Journal of Medicine*.

[20] Kimura A. (2008). Molecular etiology and pathogenesis of hereditary cardiomyopathy. *Circulation Journal*.

[21] Chang Y.-F., Imam J. S., Wilkinson M. F. (2007). The nonsense-mediated decay RNA surveillance pathway. *Annual Review of Biochemistry*.

